# Private sector, for-profit health providers in low and middle income countries: can they reach the poor at scale?

**DOI:** 10.1186/1744-8603-10-52

**Published:** 2014-06-24

**Authors:** Elizabeth Tung, Sara Bennett

**Affiliations:** 1 Johns Hopkins Bloomberg School of Public Health, 615 N Wolfe St, Baltimore, MD, USA

**Keywords:** Private for-profit, Health market, Quality of care, Scale

## Abstract

**Background:**

The bottom of the pyramid concept suggests that profit can be made in providing goods and services to poor people, when high volume is combined with low margins. To-date there has been very limited empirical evidence from the health sector concerning the scope and potential for such bottom of the pyramid models. This paper analyzes private for-profit (PFP) providers currently offering services to the poor on a large scale, and assesses the future prospects of bottom of the pyramid models in health.

**Methods:**

We searched published and grey literature and databases to identify PFP companies that provided more than 40,000 outpatient visits per year, or who covered 15% or more of a particular type of service in their country. For each included provider, we searched for additional information on location, target market, business model and performance, including quality of care.

**Results:**

Only 10 large scale PFP providers were identified. The majority of these were in South Asia and most provided specialized services such as eye care. The characteristics of the business models of these firms were found to be similar to non-profit providers studied by other analysts (such as Bhattacharya 2010). They pursued social rather than traditional marketing, partnerships with government, low cost/high volume services and cross-subsidization between different market segments. There was a lack of reliable data concerning these providers.

**Conclusions:**

There is very limited evidence to support the notion that large scale bottom of the pyramid models in health offer good prospects for extending services to the poor in the future. In order to be successful PFP providers often require partnerships with government or support from social health insurance schemes. Nonetheless, more reliable and independent data on such schemes is needed.

## Background

The “Bottom of the Pyramid” (BoP) concept suggests that the poorest segments of the population are an untapped market for goods and services, and that multinational companies providing goods and service to this population can both be profitable and aid social development [1]. Typically such companies need to pursue a strategy that combines low profit margins (and prices) with high volume in order to be successful. BoP applications to the information and communication technology sector are the best documented, and such businesses have received most attention in India.

However there has also been substantial criticism of the BoP idea. For example, critics have argued that Prahalad overestimated the number of people making less than $2 a day, and thus inflated the size of the market at the bottom of the pyramid [2]. Karamchandani et al [3] argued that few companies have been able to achieve the scale envisaged by the BoP argument. Landrum [4] questioned the transferability of the concept beyond India, where there are relatively large and concentrated, poor populations. Case studies of ICT kiosks in India that were targeted at the poor found that profit margins associated with serving this market were unattractive to private for-profit (PFP) firms, and thus de facto they served the middle class, and also were focused in urban rather than rural areas [5]. Pitta et al [6] concluded that “there is no agreement in the literature about the potential benefits of the BOP approach for both private companies and low-income consumers”.

Prahalad [1] discusses the relevance of the BoP concept to the health sector, and certainly substantive evidence points to the role that the private sector already plays in caring for the poor. According to the IFC, “In Ethiopia, Kenya, Nigeria, and Uganda, more than 40% of the people in the lowest economic quintile receive health care from private, for-profit providers” [7] (pp8). In South Asia about three quarters of children from the lowest economic quintile with acute respiratory infections seek care in the private sector [8]. There are relatively few studies however that seek to assess the role that large private-for-profit companies currently play, and may play in the future, in providing services to the poor in low and middle income countries. Bhattacharyya et al [9] is one study that goes beyond simple description of a single initiative. The study reviewed and analyzed a number of innovative private sector service delivery models and identified key characteristics of these models. However this paper was not focused on the PFP sector and many of the initiatives mixed for-profit and non-profit modalities. Common characteristics of these innovative initiatives included (i) a focus on minimizing unit costs through reducing input prices and streamlining of medical processes, (ii) high patient volumes and (iii) cross-subsidization from wealthier patients to poorer patients. Bhattacharya concluded that there was little rigorous evidence of the quality of care provided or the extent to which services really reached the poor.

## Methods

**Figure 1:**
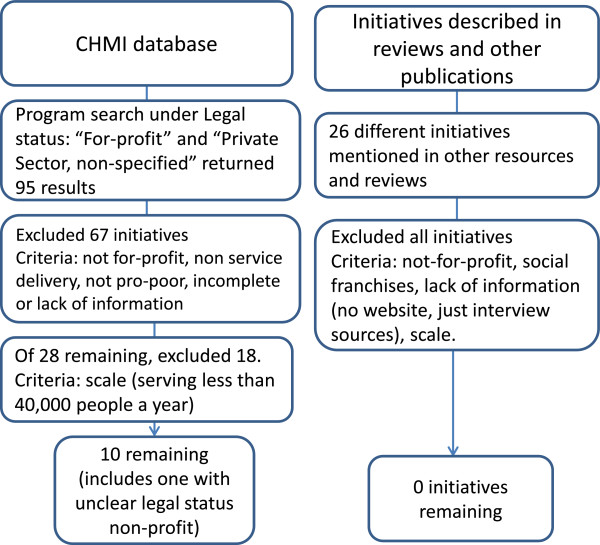
Search and selection process.

This study considers the extent to which for-profit, bottom of the pyramid models (BOP) are currently active at scale in LMICs with a view to assessing their potential in the future. Specifically we address the following questions:

1. Are there large-scale, PFP companies that provide health services for the poor?

2. Is there evidence of the impact of such PFP models on the quality and accessibility of care for the poor?

3. What are the key characteristics of BOP business models in the health sector and in particular which characteristics have enabled them to reach a large scale?The analysis is limited to initiatives that deliver health services, excluding companies that focus on private health insurance, or commodities like drugs and family planning alone. Only initiatives that have reached a large scale were included, rather than small pilots that may never manage to scale up successfully. For this report “large scale” was defined as carrying out at least 40,000 outpatient consultations a year, or representing about 15% of a type of service in a their country. The threshold of 40,000 outpatient consultations a year was used because it represented a natural “break” in the data. As noted in Figure 1, of the 28 initiatives that qualified on all other criteria, 18 were eliminated because of the scale definition. The majority of these had no information on scale. If the scale threshold had been lowered to 10,000 outpatient consultations annually, five additional initiatives would have qualified. If the threshold had been raised to 75,000 patients a year, only three companies would have been eligible.

Ethical review was not sought for this study as it was comprised solely of a desk based review of existing literature.We reviewed both published and grey literature, and adopted the following procedures:

**Figure 2:**
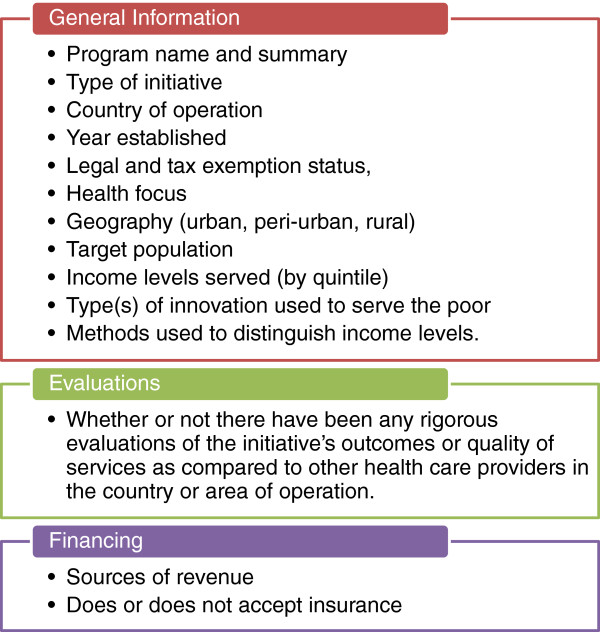
Information extracted regarding included initiatives.

i. All PFP provider initiatives contained in the Center for Health Market Innovations (CHMI) database (http://healthmarketinnovations.org/), (which is the most comprehensive resource available on private sector initiatives in low and middle income countries) were identified. A search in January 2012, returned a total of 95 results for “Private sector (for-profit)” and “Private Sector, non-specified” legal status.

ii. Detailed review of program summaries in the CHMI database, led to the exclusion of 67 initiatives because they were not for-profit, or were not related to health service delivery, or did not serve the poor, or the CHMI database indicated that the information was either incomplete or the initiative was no longer active. We did not seek to identify firms that self-identified as adopting a BoP model but rather included all companies that stated that part of their target client base included poor people.

iii. For the remaining 28 initiatives, a Google, Google Scholar and PubMed search was conducted by company name in order to gather information on scale. An additional 18 programs were excluded at this juncture either because there was very little information available on the initiative (including no company website) or because they were not considered large enough in scale, as defined previously. Information on scale was not available in a consistent format, and the numbers of patients seen per year are often estimates based on information found in the grey literature. In some cases, the number of beds in a given country was reported, and the annual number of patients served was triangulated by comparing it to similar organizations with similar bed capacity.

iv. Existing reviews of private sector initiatives (including [7,10-13]) were hand searched to identify further initiatives. This yielded 26 initiatives that were not included in CHMI database. All of these were excluded either because they were not for-profit, or were not at scale, or did not have sufficient information available through a Google® and Google scholar® search. Figure 1 summarizes the search and selection process. Additional file 1 contains a list of all the initiatives and the reason they were excluded.v. For each included company, a more in-depth search was conducted through the following: PubMed, Global Health, Embase, Scopus, Business of Healthcare, Business Source Complete, Google scholar and Lexis Nexis Academic. The type of information extracted for each initiative is summarized in Figure 2.

In reviewing the included initiatives we sought to identify and extract information concerning the technical or perceived quality of services provided, and information regarding how the initiative had affected the accessibility of care (either geographical or financial). With regard to key characteristics of BoP business models, we had no prior framework concerning which types of characteristics might be key, instead all available information on the business model was extracted, and we sought to compare across the different firms included to identify commonalities and differences in their approach.

## Results and discussion

### Overview of initiatives

Table 1 summarizes characteristics of the included organizations. Very few initiatives met all of the inclusion criteria. Seven of the ten initiatives identified are chains of clinics or hospitals, where a single company owns and operates multiple hospitals or clinics, based in different geographical areas. All of the hospital chains are based in India, except for the two eye care specialty chains – Visualiza in Guatemala and the Lumbini Institute in Nepal. The chains range in size: LifeSpring clinics have about 20 beds each, whereas Narayana Hrudayalaya’s (NH) flagship hospital has 1000 beds. Of the remaining initiatives: CEGIN is a network of private providers which agree to accept CEGIN members at reduced prices; Queen Mamohato Memorial Hospital in Botšabelo, Lesotho is a public hospital with associated filter clinics that is operated by a private consortium; and Ziqitza is a PFP emergency response service based in India. Ziqitza provides some basic clinical services inside of the ambulances, but clearly not to the same extent as the other clinics and hospitals. Lastly, all but two of the initiatives (Visualiza and CEGIN) operate principally in urban areas.

**Table 1:** Overview of companies included in the study

**Program name**	**Type of initiative**	**Country**	**Year establish-ed**	**Health focus**	**Clients served/number of beds**	**Number of facilities operated**
CARE Hospitals	Chain of hospitals or clinics	India	1997	Cardiology & other specialty services. Primary health care in urban and rural areas	450,000 outpatients, 30,000 admissions, 4,000 cardiac surgeries	12 hospitals and a number of clinics with a total of 1600 beds
Centro Ginecologico Integral (CEGIN)	Network of providers	Argentina	1989	Gynecology	40,000 patients per year	60 independent health providers in the network
Lifespring hospitals private Ltd.	Chain of hospitals or clinics	India	2005	Maternal and Child Health	About 50,000 outpatient and inpatient consultations per year	9 hospitals with 20 beds each
Lumbini Eye Institute	Chain of hospitals or clinics	Nepal	1983	Eye care	Provides 25% of eye care in Nepal, treating about 260,000 patients per year and performing 30,000 surgeries per year	1 main hospital with 215 beds, 3 secondary hospitals and a number of primary clinics
Narayana Hrudayalaya Hospital (NH)	Chain of hospitals or clinics	India	2001	Chronic diseases (heart surgeries, cancer, orthopaedics, kidney disease)	6,000 operations per year which represents about 12% of heart surgeries in India. No estimates of patient numbers were found, but NH has about 3× capacity of CARE hospitals	Currently has 5000 beds in India
Queen Mamohato Memorial Hospital	Stand alone clinic or hospital	Lesotho	2002/03	General primary, secondary and tertiary care	About 187,000 patients per year	1 referral hospital and 3 filter clinics
Vaatsalya Hospitals	Chain of hospitals or clinics	India	2004	Primary and secondary care	400,000 patients	14 hospitals
Visualiza	Chain of hospitals or clinics	Guatemala	1997	Eye care	Screens over 30,000 patients per year and performs 30% of cataract surgeries in Guatemala	
Viva Sehat (formerly Razi clinics)	Chain of hospitals or clinics	India	2009	General primary care	About 230,00 patients per year	65 clinics in Hyderabad
Ziqitza	Ambulance services	India	2005	Eye services	About 50,000 patients per year	90 ambulances

It was frequently difficult to define the legal status of the company, and in particular to understand its for-profit status. For example, when CEGIN was a purely for-profit enterprise, it appeared to only reach about 9000 people. In 2004 it established a membership card program (known as the SER program) that provides discounted access to CEGIN services. This enabled CEGIN to expand its coverage by about 5 times [14], and is the reason that it reached the scale to be included in the study. But all of the proceeds from the membership cards go to a tax-exempt foundation, called the SER Foundation.

NH and CARE were the only two companies that extended care to poorer patients in rural areas, and they did this through their charitable arms and often with the use of technology. For example, each of NH’s rural coronary care units is linked to an NH center via video-conferencing and software that enables rural staff to transmit ECG images for consultation with an NH specialist. This service is supported by the Asia Heart Foundation and is free to clients [15]. CARE Foundation actually pre-dates the for-profit hospital chain. Similarly to NH, part of its mandate has been to expand telemedicine, including installing image sharing software, so that rural patients are able to benefit from specialists who are based in urban areas. Additionally, CARE Foundation has forged a partnership with the government of India and other private foundations to pay for about 500 pediatric heart surgeries a year [16].

Partnerships or agreements with government were often critical to the success of the company. Companies that practiced cross-subsidization at a large scale attracted patients with state subsidized insurance (RSBY in India and the majority of CEGIN’s clients have state health insurance), which provides them with a base of poor clients who can still pay. NH, has gone a step further and forged an insurance product, called Yeshasvini, in partnership with the state of Karnataka. Yeshasvini provides coverage to farmers that have belonged to a cooperative for at least one year, with the state government paying the bulk of the premium [15]. CARE has stated that it hopes to also introduce its own micro-insurance product that will complement RSBY.

Queen Mamohato Memorial Hospital is another example of public-private partnership. To raise capital for a 400+ bed hospital and three filter clinics (requiring approximately $120 million), a Private-Public Investment Partnership (PPIP) was established between the Government of Lesotho and private groups. Under the PPIP, the Government of Lesotho contributed roughly 36 percent of total costs, and the remaining 64 percent came from private sources, primarily the Development Bank of Southern Africa and the Tšepong consortium. Netcare, one of South Africa’s largest private hospital groups, is the largest stakeholder (40%) of the Tšepong consortium, which is made up of a group of local and international healthcare providers. Under an 18-year agreement, Netcare will also provide all clinical and non-clinical services in the health care facilities. Netcare will generate returns on 35 private hospital beds that can serve patients with private insurance as well as by using government infrastructure, like radiology theaters. The rest of the hospital beds, however, will be general ward and open to the public, which is largely low-income in this underserved area [17].

### Characteristics: delivering value to clients

We did not find any evaluations of perceived quality of care in the included initiatives and only one study of technical quality, that examined surgical outcomes of cataract surgeries performed in the Lumbini and Bheri zones in Nepal [18]. While the study’s findings are not only attributed to work performed by the Lumbini Eye Institute, its findings that both clinical and visual functioning or quality of life outcomes were below expected levels demonstrates the importance of evaluating outcomes. For heart surgeries, NH has reported an overall hospital mortality rate of 2% and a hospital-acquired infection rate of 2.8 per 1000 ICU days, but it seems that this data has not been published in a peer reviewed journal [19]. Other sources have cited that CARE has comparable outcomes for heart surgeries without reporting specific figures [20]. Overall, information on health outcomes is not systematically available.

The majority of the companies are based in urban or peri-urban settings, which means that they are in densely populated areas with many potential customers. Many of the hospital chains are located in smaller cities. From the start, Vaatsalya focused on small and medium towns that lacked secondary health care services, its strategy was to create small hospitals that could fill this gap and reduce travel-related health expenses [21]. Similarly, LifeSpring was founded to serve low to middle income clients in peri-urban areas that were not satisfied with public hospitals and could not afford the existing expensive private hospitals [22]. CARE hospitals, which are larger than Vaatsalya and LifeSpring facilities, also chose to locate in lower income peripheral urban areas, in order to be closer to their target population, and not compete with private hospital groups like Fortis or Apollo that are located in metropolitan centers and target high income individuals [20].

Few of the companies emphasized traditional marketing techniques but instead carried out community outreach. This approach may be particularly suited to low income populations because its educational component explains why and when it is necessary to seek care at a health facility [6]. CARE trains Village Health Champions to provide health information and algorithm-based guidance on whether or not medical care should be sought. The Champions refer people to CARE facilities if needed [16]. Ziqitza conducted outreach efforts to hospitals and policemen to encourage them to refer patients to their service, as well as use it themselves to transport patients [23]. LifeSpring outreach workers hold monthly health camps in the hospital catchment area to raise publicity and explain why women should deliver in a hospital [22].

For poor clients, personal contact and an ensuing dialog is more likely to capture and keep customers [24]. In order to persuade poorer and more conservative consumers, it is also important to target groups or social networks, since they “lower risk by shopping together and comparing notes”, as well as offer samples or product demonstrations [24]. LifeSpring uses both of these strategies. Firstly, if it identifies pregnant women during its monthly outreach camps, its outreach workers give her a voucher for some services at the center to encourage her to visit a LifeSpring facility and try it. Additionally, LifeSpring offers (unspecified) loyalty rewards to existing patients for each word-of-mouth referral, and 90% of its customer base has been referred by a personal contact [22].

All of the initiatives state that their superior customer orientation, as compared to government hospitals, is the main reason that they are able to attract patients. Examples of being sensitive to the needs of target populations include locating facilities closer to lower income populations, having longer opening hours to accommodate people’s working schedules, and appointments rather than waiting lines. Many of the initiatives also emphasized creating a culture of respect for the patient. For example, LifeSpring has a customer CARES protocol (Courteous, Attentive, Respectful, Enthusiastic and Safe), which all staff members are expected to follow [22]. Vaatsalya routinely carries out customer satisfaction surveys to ensure that its patients feel “cared” for by its medical staff [25].

Careful attention to the preferences and purchasing habits of target populations has led to innovations in the pricing of services and payment modalities. Private facilities fulfill patients’ aspirational preferences by offering basic but clean facilities and options for more privacy. The hospitals and clinics generally offer three types of rooms with tiered pricing: private, semi-private and general ward. To alleviate the anxiety associated with paying for health care, Vaatsalya emphasizes having very clear and transparent billing to enable its clients to verify all charges [25]. LifeSpring prominently advertises its bundled services for Caesareans and vaginal deliveries that have an all-inclusive price [22]. Low-income customers tend to buy goods or services in smaller quantities and more frequently because their incomes vary more. To accommodate this CEGIN uses micro-credit to break down the cost of more expensive procedures into manageable payments. Members can access a loan from CEGIN’s micro-credit fund if they require a more expensive procedure [14].

### Capturing value: profits

Since the initiatives are pro-poor, most of them use specialization and standardization to reduce costs and achieve high patient volumes. Only Viva Sehat, which has 65 clinics throughout Hyderabad, provides broad primary care, but there is very little information currently available on it. Specialized services are more amenable to a smaller number of standardized diagnosis and treatment protocols. The use of protocols is assumed to lead to good outcomes and the avoidance of unnecessary and costly complications and medical procedures. For example, LifeSpring hospitals immediately refer all complicated cases to larger hospitals. Only taking on simpler cases for which there are protocols in place, allows LifeSpring to employ clinical staff with less experience and lower wage expectations [3]. According to its program description in the CHMI database, Viva Sehat provides doctor consultation and diagnostic services according to software-based standardized treatment protocols. They also maintain electronic patient profiles in order to minimize errors and enable quality follow-up of patients.

Some groups reach high volumes by focusing on relatively simple and low cost services. CEGIN, in Argentina, focuses on delivering gynecology services to low-income women, especially cervical cancer screening. Private doctors agree to participate in this network and offer services at reduced prices, because they gain a higher volume of patients [26]. Aravind, which was not included in this analysis because it is now a charitable trust rather than a for-profit model, pioneered the specialty care system at high volumes in low-income settings. Its method, which has been documented extensively elsewhere, achieves its volumes by conducting surgeries in a production line [27].

Part of the reason that this model works for Aravind and similar groups like Lumbini and Visualiza, is that cataract surgery is a short intervention that does not require substantial follow-up or rehabilitation. Other groups like NH and CARE, which both focus on chronic diseases and began by emphasizing cardiac care, show that the same approach can be adapted to heart surgery. According to the Wall Street Journal, Dr. Shetty of NH has called his strategy “the Wal-martization of healthcare”, and his facilities perform approximately 19 open heart surgeries and 25 catheterization procedures a day, which is about eight times higher than the average Indian hospital.

The most common characteristic of initiatives is that they focus on minimizing costs. Many of the initiatives avoid costly infrastructure investments. For example, CEGIN does not require its own infrastructure since it is a network of providers. All of the hospital chains based in India, except for NH, lease rather than own the land or buildings they operate in. Their locations in peri-urban areas or in Tier 2 and 3 towns mean that real estate is less expensive. NH differs in that it is located in Bangalore and has built its own facilities, but this was done with a subsidy from within the family [15].

Most of the companies also aggressively reduce costs for fixed assets and supplies. Some of the larger initiatives leverage their size to negotiate favorable agreements with manufacturers. For example, in addition to using generic drugs, NH negotiates short-term contracts with manufacturers to routinely get supplies at 30-35% lower cost [15]. NH and other groups also maximize the use of fixed assets in order to lower unit costs. For example CARE hospitals use their radiology equipment throughout the day for outpatient appointments, and at night for inpatients [16].

The smaller hospital chains avoid investing in expensive equipment or capacities by simplifying what they offer. LifeSpring Hospitals, which focuses on maternal and child health, mainly offers outpatient services through its network of small hospitals (25-30 beds), with deliveries as its only inpatient service. The hospitals do not have food services or their own emergency transport fleet, but depend on other state and non-governmental services [28]. LifeSpring and Vaatsalya Hospitals also do not have their own blood banks or laboratory facilities. Instead, their facilities are strategically located near larger facilities, including medical schools, that do have these capacities, and they either refer patients to these services or establish agreements to use these resources rather than invest in their own [22,25].

Lastly, the companies exhibit innovative strategies to reduce human resources costs. LifeSpring and CEGIN tend to hire recent clinical graduates who are less experienced and are willing to work in small private hospitals to gain experience. CARE has its own training program to enable task-shifting, where physician assistants take on the work of residents. This provides physicians more time for research, which helps keep them satisfied and retains them [20]. At NH, doctors are paid fixed salaries that are comparable to other private hospitals, but they are expected to work longer hours and perform more procedures. This approach allows NH to spend 22% of its revenues on salaries compared to 60%, which is commonly found in the West [15].

Many of the companies studied reach the poor through cross-subsidization that is using higher mark-ups on wealthier patients to partly subsidize care for poorer patients. CARE states that 70% of its patients are subsidized to varying degrees or do not pay; NH up to 60%; Ziqitza, 20%; and Lumbini, 12%. This information was not available for Visualiza, CEGIN or LifeSpring. NH prints daily profit and loss statements in order to know in real time the balance that they need to strike. Free procedures will be postponed in order to ensure that the company maintains a healthy bottom line [21].

Some of the companies, do not use cross-subsidies, but instead, because of their locations they end up serving low income populations. For example, Queen Mamohato Memorial Hospital has 395 general ward beds and 35 private ones, but the private rooms are used to generate profits for Netcare rather than to extend care. Vaatsalya recognizes that its prices are still out of reach for the poorest quintile, but it would not able to expand or survive if it were to reduce its prices further. Similarly, Viva Sehat does not offer tiered pricing, though there is not documentation describing why.

## Conclusions

This analysis showed that most for-profit companies reaching our measure of scale are based in urban and peri-urban areas in South Asia, and that there are very few of them. Since most companies follow the BoP model of low-cost, high-volume services, it makes sense that the companies are based in areas with high population density. Further the mix of incomes means that they can cross-subsidize between patients. Even so, many of the for-profit companies we examined used charitable arms or partnerships with the government to expand their services to the poor.

Bhattacharaya et al [9] found many similar practices to those in our study, including social rather than traditional marketing, partnerships with government, low cost/high volume services and cross-subsidization. However eight of ten of the innovative organizations studied by Bhattacharaya were non-profit. By focusing solely on for-profit companies our review sheds light on some of the practices that contribute to their survival and growth – and they are surprisingly similar to those pursued by non-profits. None of the companies included here used traditional marketing techniques, instead using community outreach and education. All focused on improving their customer orientation in a number of ways, including decreasing opportunity costs of seeking care through more convenient locations and suitable opening hours; treating clients respectfully; and bundling services with transparent prices. Medically, the companies minimized their costs: some leveraged their size to reduce input costs while others simplified the services they offered, and most had strategies to make human resources more affordable. All of the companies standardized their medical processes as much as possible.

This study’s most challenging limitation was the availability of documentation. It was often difficult to find accurate indicators of scale which may have affected the initiatives’ inclusion. Further, our cut-off point regarding scale, (ie. excluding companies serving fewer than 40,000 clients per year), though indicated by a natural break in the data, was arbitrary, and it is possible that including somewhat smaller firms may have enriched the evidence. Data on prices and profit margins were also not available. As a desk review, the analysis depended on a limited number of case studies that have been written by various business schools and organizations over the last ten years, as well other grey literature. Therefore the findings of this landscape analysis may be biased towards companies that had more documentation. Information was frequently incomplete, or had not been subject to fact checking or peer review. There is also a bias towards English language resources, which may underrepresent companies working in Latin America, Francophone Africa and Asia.

Two main policy implications emerge. First, there is still a dearth of empirical evidence on the relevance of the Base of the Pyramid concept in the health sector. In particular, evidence regarding quality of care is needed, but also the extent to which large for-profit companies actually reach the poor rather than the middle class. The majority of companies included offered specialized care. Broad primary care services may typically have lower margins and would require a longer time to profitability. Viva Sehat clinics were the exception, but there was very limited information on the company to shed light on what made it successful.

Second, the Prahalad vision of multinational companies serving the poor does not appear to have materialized in the health sector. Instead, a host of home-grown private companies are serving the BoP through partnerships with foundations and government, and non-profit organizations are probably more active than for-profit firms in serving the poor. Risk-sharing schemes, appear to be a more promising way governments and donors can engage with for-profit health care companies to reach the poor on a larger scale. This may especially be the case in areas where there is a lower population density and smaller middle class that can help support for-profit hospital or clinic chains.

## Abbreviations

BoP: Bottom of the Pyramid; CEGIN: Centro Ginecologico Integral; CHMI: Center for Health Market Innovations; ECG: Electrocardiogram; ICU: Intensive Care Unit; LMIC: Low or Middle Income Country; NH: Narayana Hrudayalaya’s; RSBY: Rashtriya Swasthya Bima Yojana (Hindi for National Health Insurance Program, India).

## Competing interests

The author’s declare that they have no competing interests.

## Author’s contributions

ET and SB jointly conceptualized the paper. ET conducted the literature search and data extraction. ET and SB jointly drafted the paper and both have reviewed and approved the final manuscript.

## Supplementary Material

Additional file 1 List of excluded initiatives. Click here for file
