# Supplementary material for: Private sector, for-profit health providers in low and middle income countries: can they reach the poor at scale?
Source: Global Health. 2014 Jun 24;10:52. doi: 10.1186/1744-8603-10-52 (PMC4094686; doi:10.1186/1744-8603-10-52)
Supplement: Additional file 1 — List of excluded initiatives. [file 1744-8603-10-52-S1.docx]

**Annex 1 – List of excluded initiatives**
